# Mango Dermatitis After Urushiol Sensitization

**DOI:** 10.5811/cpcem.2019.6.43196

**Published:** 2019-09-30

**Authors:** Michael J. Yoo, Brandon M. Carius

**Affiliations:** Brooke Army Medical Center, Department of Emergency Medicine, San Antonio, Texas

## Abstract

Prior exposure to poison ivy and poison oak, which are plants in the Anacardiacea family and contain high levels of urushiol, appear to be a risk factor for delayed hypersensitivity reactions to mango fruits. Cross-sensitization between these plants and mangos is believed to be secondary to an overlap in the urushiol antigen and 5-resorcinol, found predominately in mango peels. This unique combination of sensitization and reaction constitutes a type IV hypersensitivity response, mediated and driven by T cells reacting to similar antigens. We present a case of an otherwise healthy man, with a remote history of poison ivy exposure, who presented with a delayed but significant reaction to mango fruit. Obtaining the patient’s history of prior plant exposures and reactions was key to isolating the likely underlying causation of his presentation.

## INTRODUCTION

Urushiol is an allergenic substance found in the Anacardiaceae family, most commonly known for poison ivy (*Toxicodendron radicans*) and poison oak (*T. diversilobum*).^1^ Presentations of patients who come in direct contact to urushiol-containing plants include pruritus, erythema, vesicles, bullae, and localized edema. While systemic reactions like anaphylaxis can cause airway compromise, the burning and inhalation of urushiol-containing plants can cause acute airway complications such as tracheitis and pulmonary edema.^2^ Although contact dermatitis and airway reactions are well documented in the literature, a lesser-known reaction from prior urushiol exposure is hypersensitization to mango fruits. We present a case of a 41-year-old man with suspected mango dermatitis, incited by mango handling after remote exposure to poison ivy.

## CASE REPORT

An otherwise healthy 41-year-old man presented to the emergency department (ED) with a severely worsening, four-day-old diffuse, pruritic rash, which began in the inguinal regions bilaterally but had since spread to his trunk and extremities. The patient’s primary complaint was insomnia secondary to his pruritis. He initially denied any new medications or other exposures but did endorse a distant episode of contact dermatitis to poison ivy two years prior. On arrival, his vitals included temperature of 98.3º Fahrenheit, blood pressure of 133/87 millimeters of mercury, heart rate of 69 beats per minute, respirations of 18 per minute, and a pulse oximetry of 100% on room air.

The patient’s physical exam was remarkable for a macular, blanching, non-vesicular, erythematous rash on all extremities, chest, and back, sparing the palms, soles, and oral mucosa. Lungs were clear to auscultation in all fields. Further diet history detailed consumption of two mangos two days prior to the onset of the rash. The patient’s wife, who accompanied him to the ED, had also consumed mangos two days prior but was asymptomatic. Although both the patient and his wife handled the mango peels, the wife did not endorse prior plant exposures resulting in rash.

Given the patient’s substantial discomfort, intravenous (IV) access was established, and 50 milligrams (mg) of diphenhydramine and 50 mg ranitidine were parenterally administered. This resulted in significant relief of pruritus and mild improvement in visible rash. After a brief observation period, the patient was discharged home on 60 mg of oral prednisone for five days and 20 mg of oral loratadine, as needed. Of note, the patient deferred the first steroid dose in the ED due to the evening time of presentation and a remote history of insomnia after taking steroids. He was contacted five days after his ED visit with almost complete resolution of symptoms and significant improvement in insomnia with over-the-counter (OTC) oral diphenhydramine. Extending steroid treatment for an additional week was discussed, but the patient declined based on the improvement of his symptoms. He was contacted again approximately three months after his ED visit and denied any rebound symptoms.

## DISCUSSION

The classic hypersensitivity framework of the Gel and Coombs system defines four main classes of reactions: types I-IV. In brief, type I reactions are mediated by antigens cross-linking immunoglobulin (Ig) E, causing mast cells and basophils to release histamine and other vasoactive contents.^3^ Type I responses range from seasonal allergies to asthma to the extreme of anaphylaxis. Type II reactions are predominately mediated by IgG and IgM, stimulating phagocytes and natural killer cells to either uptake IgG and IgM tagged antigens or active complement.^3^ Examples of type II responses include hemolytic anemia and basement membrane disease. Type III reactions are also mediated by IgG but cause pathology by the formation of immune complexes.^3^ These complexes deposit around small vessels and tissues and can manifest in diseases such as systemic lupus erythematosus and glomerulonephritis. Finally, type IV reactions are T-cell mediated. After initial sensitization to an antigen, T-cells release damaging cytokines upon subsequent exposure to structurally similar antigens.^4^ Unlike types I-III, type IV responses do not involve Igs. These responses can manifest from contact dermatitis to Stevens-Johnson syndrome.^4^

Urushiol is a well-known hapten to skin proteins that induces a type IV hypersensitivity response.^2^ After initial sensitization to urushiol, typically from contact with poison ivy or poison oak, subsequent exposures to urushiol produce a cell-mediated memory response after two to three days.^2^ Limited studies demonstrated a cross-hypersensitivity response between urushiol and the mango compound, 5-resorcinol, found predominantly in the skin, leaves, and stems of mango fruits.^5^ 5-resorcinol, along with other phenols, are collectively known as “mango latex,” which acts as a preservative with anti-microbial properties.^5^ Hershko et al. identified that mango pickers with severe rashes had prior exposures to poison ivy or poison oak when compared to pickers with mild or no rashes working in the same conditions.^6^ Interestingly, these allergens appear to be negligible in the actual fruit of the mango, and patients with a history of mango dermatitis may still enjoy the fruit if peeled by another person.^7^ Although the pathophysiology of this cross-reaction is not well described, understanding this phenomenon is important due to the abundance of mango fruits worldwide.

Management of mango dermatitis and contact dermatitis from poison ivy or poison oak is nearly identical and primarily entails avoidance of inciting factors and symptomatic treatment. Post-exposure, patients should be advised to gently rinse the affected area with cold, soapy water, ideally within 30 minutes, to minimize dermal absorption. Cool compresses and calamine lotions are OTC options for symptomatic management.^1^ Adjunct systemic corticosteroids are indicated in moderate to severe dermatitis, typically dosed at 1 mg per kilogram per day (kg/day) for 14–21 days.^1^ Although the length of corticosteroid therapy is not extensively studied, the prolonged course is recommended over shorter courses to prevent rebound dermatitis.^8^

**CPC-EM Capsule**

What do we already know about this clinical entity?

*Urushiol is an allergen of poison ivy and poison oak. Prior exposure to it can cause a type 4 hypersensitivity response upon subsequent handling of mango peels*.

What makes this presentation of disease reportable?

*We describe a delayed, nonspecific dermatitis whose etiology was discovered only upon obtaining a detailed history of prior allergic reactions to poison ivy*.

What is the major learning point?

*Patients who previously experienced hypersensitivity reactions to poison ivy or poison oak are at risk for hypersensitivity reactions when handling mango peels*.

How might this improve emergency medicine practice?

*When approaching an undifferentiated rash, an accurate history of prior reactions and allergies can help identify the etiology of the rash*.

Of note, atopic dermatitis (a type I hypersensitivity response) and contact dermatitis (a type IV hypersensitivity response) can present similarly, and understanding the differences between the two can guide treatment, such as the use of antihistamines, and identifying inciting factors. This patient was given IV antihistamines on arrival, to provide symptomatic relief for a presumed, undifferentiated allergic reaction that disrupted the patient’s sleep. Based on our current understanding of pruritus, especially in non-histamine mediated pruritus (i.e., contact dermatitis), the use of either IV or oral antihistamines has limited evidence for use. However, antihistamines can provide a sedating effect that may be useful in select patients who are unable to sleep secondary to extreme pruritus.^9^

## CONCLUSION

Rashes are common presentations to the ED. While most are non-anaphylactic and therefore generally non-emergent, key dietary and exposure histories are helpful in determining the etiology and treatment of undifferentiated rashes in the ED setting. Clinicians should recognize that cross-reactions between allergens are frequent and remain suspicious about cross-hypersensitivity reactions in any patient with a known history of allergic reactions.
